# Recognizing an Abnormal Course of the Temporal Division of the Facial Nerve

**Published:** 2014-07-23

**Authors:** Sami P. Moubayed, Daniel A. Barker, Jeffrey Rawnsley, Keith E. Blackwell, Gregory S. Keller

**Affiliations:** ^a^Division of Otolaryngology—Head and Neck Surgery, Université de Montréal, Montreal, Canada; ^b^Department of Head and Neck Surgery, David Geffen School of Medicine at UCLA, Los Angeles, Calif

**Keywords:** facial nerve, temporal division, trajectory, fascial relationships, abnormal

## DESCRIPTION

**Figure 1:**
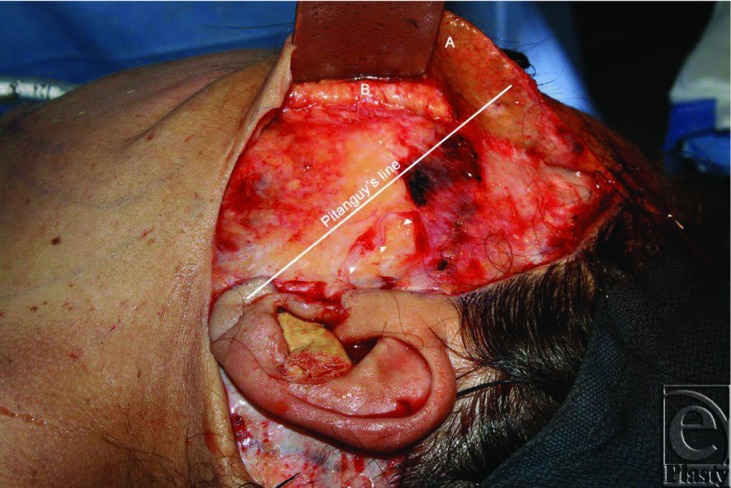
The temporal division of the facial nerve (arrowheads) is seen having an abnormal course over the zygomatic arch (arrows) beneath the elevated skin (*a*) and SMAS (*b*).

A middle-aged woman was undergoing elective rhytidectomy by the senior author using a high superficial musculoaponeurotic (high-SMAS) approach deep plane facelift. After horizontal SMAS division and elevation over the zygomatic arch, the temporal division of the facial nerve was seen crossing the zygomatic arch approximately 1 cm anterior to the tragus with an initial vertical trajectory before coursing anteriorly (Fig 1). The facial nerve emerged posterior to its reported course in the literature and became superficial inferior to what is reported in recent literature articles.

Injury to the temporal branch of the facial nerve is one of the most undesirable surgical outcomes in the temporal region. Its course is most commonly described as running along Pitanguy's line, although deviations from this trajectory are seldom discussed among clinicians. Moreover, conflicting data have been written about its fascial relationships over the years. Given its vulnerability during extended aging face procedures, as well as surgical approaches to the temporomandibular joint, maxillofacial trauma, or temporal artery biopsy, a thorough understanding of the temporal branch anatomy in terms of trajectory and fascial planes is essential to avoid iatrogenic injury.

## QUESTIONS

1. **What is the classic trajectory of the temporal division of the facial nerve?**
2. **In what instances is this trajectory defined as abnormal?**
3. **What are the fascial relationships of the temporal branch in the temporal region?**
4. **What are the clinical correlates of these relationships?**

## DISCUSSION

Several anatomical studies have described the trajectory of the temporal division of the facial nerve after its emergence from the parotid.^1^^-^5 The most commonly reported course runs along what is termed Pitanguy's line, which was described by Pitanguy and Ramos in 1966,^4^ and courses between a point 0.5 cm below the tragus and a point 1.5 cm above the lateral eyebrow. The trajectory in our patient significantly deviates from Pitanguy's line.

To understand the normal anatomical variations in its trajectory, multiple dissection studies have dissected the temporal branch of the facial nerve. We have reported a select group of landmark studies in Table 1 describing the course of the temporal branch of the facial nerve in terms of the location of its most posterior branch, as well as its main trajectory. The closest position to the external auditory canal of the temporal branch has been described as being 8 mm, but most descriptions place this position significantly medial to this. In terms of trajectory, the most extensive anatomical study to date on 300 hemifaces reports it as lying between 2 lines: a line passing through the superior tragus and the uppermost forehead crease and a line passing through the inferior tragus and the lowermost forehead crease.^5^ The aberrant branch that we describe is medial to this point. One or 2 branches are commonly found at the zygomatic arch, although 3 or 4 branching patterns are possible.^5^

Much has been written on the fascial relationships of the temporal branch of the facial nerve in the temporal region. The most recent high-quality evidence has consistently shown that the temporal branch travels deep and transitions into a sub-SMAS plane before entering the muscles in the frontal region.^6^ This transition point has been termed the fascial transition zone, which occurs with 95% confidence interval 0.9 to 1.4 cm posterior to the orbital rim, and 1.5 to 3.0 cm above the zygomatic arch.

The mastery of these relationships is essential to surgeons performing common procedures such as temporal artery biopsy, facelift, temporomandibular join surgery, maxillofacial trauma, or pterional neurosurgical approaches, among others. Understanding the normal and abnormal anatomy of the temporal branch of the facial nerve in terms of trajectory and facial layers is essential for the surgeon operating in the temporal region to avoid iatrogenic nerve injury.

## Figures and Tables

**Table 1:** Selected anatomical studies describing the trajectory of the temporal branch of the facial nerve

Author	Year	Hemiface Count	Most Posterior Branch	Trajectory	Abnormal Course
Furnas	1965	115	Crosses superior border of zygoma up to 5 mm posterosuperior on a line perpendicular from hairline	Towards the lateral eyebrow	>1 cm posterosuperior from intersection of hairline and superior zygoma, and >2 cm above lateral eyebrow
Pitanguy and Ramos	1966	20	Not defined	On a line from 0.5 cm below tragus to 1.5 cm above lateral eyebrow (*Pitanguy's line*)	Not defined
Correia and Zani	1973	100	Not defined	*Pitanguy's line*	Outside two lines: 1. Superior: earlobe to highest forehead crease 2. Inferior: earlobe to lateral eyebrow
Al-Kayat and Bramley	1979	54	2.0 ± 0.5 cm anterior to external auditory canal over zygomatic arch	Not defined	Most posterior branch crosses 0.8 to 3.5 cm anterior to external auditory canal
Zani	2003	300	Not defined	Courses between two lines: 1. Superior: upper tragus to highest forehead crease 2. Inferior: lower tragus to lowest forehead crease	Anything outside the superior and inferior lines
